# Autologous Minimally Invasive Cell-Based Therapy for Meniscal and Anterior Cruciate Ligament Regeneration

**DOI:** 10.1155/2021/6614232

**Published:** 2021-06-24

**Authors:** Pradeep V. Mahajan, Swetha Subramanian, Siddhesh C. Parab, Sanskruti Mahajan

**Affiliations:** ^1^StemRx Bioscience Solutions Pvt. Ltd., Navi Mumbai, India; ^2^Department of Surgery, Indianapolis University School of Medicine, USA

## Abstract

The meniscus is a fibrocartilaginous tissue that acts as a “shock absorber,” along with performing functions such as stabilization and lubrication of the joint, proprioception, and load distribution. Sudden twisting movements during weight bearing or trauma can cause injury to the menisci, which leads to symptoms such as pain, swelling, and difficulty in performing movements, among others. Conventional pharmacological and surgical treatments are effective in treating the condition; however, do not result in regeneration of healthy tissues. In this report, we highlight the role of cell-based therapy in the management of medial and lateral meniscal and anterior cruciate ligament tears in a patient who was unwilling to undergo surgical treatment. We injected autologous mesenchymal stem cells obtained from the bone marrow and adipose tissue and platelet-rich plasma into the joint of the patient at the area of injury, as well as intravenously. The results of our study corroborate with those previously reported in the literature regarding the improvement in clinical parameters and regeneration of meniscal tissue and ligament. Thus, based on previous literature and improvements noticed in our patient, cell-based therapy can be considered a safe and effective therapeutic modality in the treatment of meniscal tears and cruciate ligament injury.

## 1. Introduction

The meniscus is a fibrocartilaginous tissue that provides structural integrity to the joint during tension and torsional movements. In addition, the meniscus functions to stabilize and lubricate the joint, along with aiding in proprioception and load bearing [1]. In the knee, the lateral and medial menisci are semilunar or “crescent-shaped” structures, which function to disperse the friction in the knee joint. These menisci act as a cushion between the femur and tibia; therefore, these are rightly called “shock absorbers.” Degenerative meniscal injuries occur in the absence of trauma, commonly in the elderly due to gradual wear and tear of tissues. On the other hand, traumatic injuries can occur at any age, commonly due to excessive pressure or sudden rotational movements of the knee joint [1]. These injuries are frequently accompanied by cruciate ligament injuries. The anterior and posterior cruciate ligaments control the back and forth movements and provide rotational stability to the knee. Therefore, meniscal and cruciate ligament tears are common in sportspersons; however, sudden daily activities/jerky movements of the knee may also lead to such injuries at any age.

Meniscal and cruciate ligament tears may go unnoticed in the immediate period following injury. Patients may feel a “pop” in the affected area; however, they may be able to continue with their activity. Gradually, over 2-3 days, swelling and stiffness may develop followed by pain, catching and locking of the knee, buckling sensation, and restricted ranges of motion.

Magnetic Resonance Imaging (MRI) is the standard tool to assess the extent of meniscal injury. Tables 1 and 2 present the grading of the conditions.

Conventional treatment of meniscal and cruciate ligament tears depends upon factors such as age, systemic condition of the patient, extent of tear, and duration predicted for the injury to heal, among other factors. Initial treatment involves icing, anti-inflammatory medications, muscle strengthening exercises, and rest. More severe cases of meniscal injury require surgical management, namely, meniscectomy, meniscal repair, and meniscal reconstruction [1]. These procedures are frequently performed in combination with cruciate ligament repair or reconstruction. Arthroscopy is a minimally invasive surgical procedure, which facilitates precise trimming or repair of the tear. Due to its minimally invasive nature, arthroscopic procedures are associated with better healing and preservation of healthy joint tissue. However, currently, neither open nor arthroscopic total meniscectomy is indicated, due to the chance of development of early osteoarthritis. Arthroscopic partial meniscectomy is frequently performed, particularly in patients with degenerative tears. Nonetheless, although short-term clinical results of the procedure have been positive, the rate of progression to osteoarthritis in the long term has also been reported [1]. Similarly, repair and reconstruction of the cruciate ligaments can be accomplished by techniques such as suturing the torn ligament, tunnel placement, and reconstruction with auto-, allo-, or xenografts. However, despite their high success rates, these procedures are associated with risks such as persistent pain, stiffness, loss of range of motion, and graft failure [2].

Considering the aforementioned disadvantages and the issue that surgical therapies do not regrow healthy tissues, a novel therapeutic option in the form of regenerative medicine and cell/growth factor-based therapy is being explored to achieve regeneration of meniscal tissue and cruciate ligaments in a minimally/noninvasive manner. Orthopedic conditions and sports injuries particularly benefit from the self-renewal, multidifferentiation ability, and other paracrine properties of stem cells and growth factors in the human body.

In this study, we report a case of high-grade medial and lateral meniscal tear along with anterior cruciate ligament (ACL) tear in a young woman, successfully treated with autologous cell- and growth factor-based therapy.

## 2. Case Presentation

A 31-year-old woman suffered a road traffic accident before 1 year, during which she injured her left knee. Since then, the patient had occasional knee pain that would increase on stair climbing. She underwent an MRI, which revealed a grade III tear of the medial and lateral meniscus and a high-grade ACL tear. The patient was also overweight, which added to her discomfort in performing daily living activities. She was prescribed anti-inflammatory medications and advised physiotherapy rehabilitation; however, she did not achieve complete recovery. The family was skeptical about the outcomes of surgical management, which was advised to the patient, considering her age and overweight status.

At our center, following consultation and thorough investigations, the patient was explained in detail about autologous cell-based therapy, and the family consented to the treatment. The patient was advised a holistic protocol of cell- and growth factor-based therapy, diet modifications, exercises, and nutraceuticals.

Prior to treatment, written informed consent was obtained from the patient and she also consented towards publication of this paper.

The patient underwent two sessions of cell- and growth factor-based therapy over a period of 1 month, with an interval of 15 days between the sessions. The source of autologous mesenchymal stem cells (MSCs) and platelet-rich plasma (PRP) was from the bone marrow, adipose tissue, and peripheral blood for the first session. Approximately, 100-150 mL of bone marrow from the iliac crest, 50-100 mL adipose tissue from the gluteal region, and 50 mL peripheral blood from the cubital vein were obtained. For the second session, only peripheral blood (50 mL) was obtained from the contralateral side (compared to the first session) as a supportive treatment to enhance healing and the function of the cells administered during the first session.

A standard protocol is followed at our center for isolation of MSCs and PRP from the bone marrow, adipose tissue, and peripheral blood samples, the summary of which is as follows. The sample obtained from the bone marrow is subjected to sedimentation followed by Ficoll density-gradient centrifugation. After a series of supernatant aspiration and saline washing to eliminate the coexistent/undesirable cells, the final pellet is obtained containing the isolated MSCs. Likewise, the adipose tissue sample is centrifuged and the fatty layer is discarded. Similar to the bone marrow procedure, saline washing and centrifugation are performed to obtain the final pellet containing the isolated MSCs. The peripheral blood sample is centrifuged at 800 rpm for 10 minutes, followed by aspiration of the superficial layer and repeat centrifugation and isolation of the platelet concentrate.

Characterization of the confluent/isolated mesenchymal stem cells is done by flow cytometry of representative bone marrow and adipose tissue samples at regular intervals at our center. The samples obtained from the representative bone marrow and adipose tissue were positive for CD90, CD73, and CD105 (all ≥ 95%), which are markers of mesenchymal stem cells.

Aspiration and transplantation of therapeutically effective dose calculation were based on grade of the condition and body mass index of the patient. 500‐5000 × 10^6^ bone marrow-derived MSCs, 1600‐400 × 10^6^ adipose-derived MSCs, and 1.5 × 10^6^ platelet concentrate were transplanted in the affected area. Intraarticular and intravenous routes were chosen for transplantation of the cells [3].

Active physiotherapy rehabilitation and lifestyle modifications were initiated 24 hours after the procedure, after each session. Being a minimally invasive procedure, the patient was discharged on the next day after each session, following thorough assessment of vitals and general condition. She was instructed to continue physiotherapy at home.

**Figure 1:**
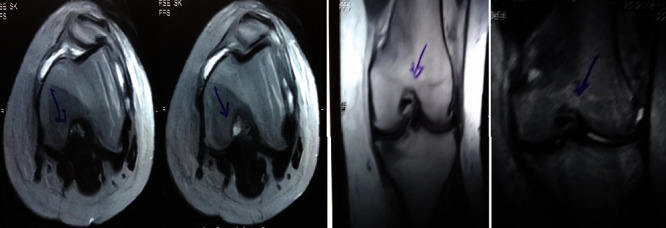
Pretreatment MRI showing grade III tear in the posterior horn of the medial and lateral menisci.

**Figure 2:**
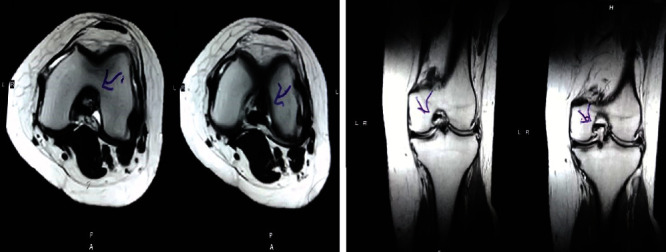
Posttreatment MRI showing improvement in the tear in the posterior horn of the medial and lateral menisci. The patient experienced symptomatic improvement as well as enhanced ability to perform her activities of daily living.

Follow-up was done one month after the therapies. The patient had experienced significant pain relief after the first session of cell-based therapy. Following the second session, she had no pain and noticed improvement in ranges of motion. An increase in strength of the lower limb muscles was also observed with continued physiotherapy. From the start of treatment (before 11 months) to the last follow-up (before four months), the patient lost 11 kilos by following the advised diet and exercise-based protocol. Consequently, she now enjoys overall good health and is able to perform her daily living activities more comfortably.  Table 3 shows the MRI findings before and after treatment. Figures 1 and 2 show the pre- and posttreatment (after 1 year) picture of the meniscal tear. Improvement in the grade of injury is observed in Figure 2.

## 3. Discussion

Extensive injury or improper treatment of meniscal tissue damage increases the risk of osteoarthritis. Arthritic changes pose a major therapeutic challenge, especially in young individuals, considering the lack of effective surgical and rehabilitation therapies. Although conventional modalities, such as partial meniscectomy, aim to preserve healthy meniscal tissue and delay the onset of osteoarthritis, the end achievement is repaired, as opposed to regeneration of tissues. Other similar therapies such as conduit treatment and abrasion therapy, among others, are also not definitive modalities for the treatment of meniscal tears. Chew et al. called stem cells the “holy grail” of meniscal tissue repair [4]. Recently, injection of PRP and meniscus tissue engineering has shown promise in the treatment of the condition [5]. Tissue engineering involves the use of cells, signaling factors, and scaffolds with regenerative potential to augment the healing process following injury. Articular chondrocytes, meniscal fibrochondrocytes, and MSCs have been used in meniscal tissue repair with promising results [6, 7]. Likewise, studies have investigated the properties of MSCs and PRP in cruciate ligament injuries, with or without the use of scaffolds. Of note is the study that reported the 2-year outcomes of a bovine-derived extracellular matrix scaffold (seeded with autologous blood to deliver growth factors) to augment primary ACL repair. The trial did not report any failures, and the morphology of the healed ligament was similar to the contralateral intact ACL in all patients [8]. The control arm used in the aforementioned trial was patients undergoing ACL reconstruction with hamstring tendon autograft; however, the results of the regenerative approach were superior.

In the present case, we employed an autologous cell- and growth factor-based approach in the management of high-grade meniscal tissue and ACL tears. We utilized autologous MSCs and growth factors; however, other studies have also reported synovial membrane, contralateral healthy meniscal tissue, and extra-articular tissues such as dermis as a source of MSCs [6, 7, 9]. Adult MSCs are particularly attractive due to their potential for multilineage differentiation, immunomodulation, and ability to migrate towards sites of injury. The procedure, being minimally invasive, enables the patient to resume routine activities within a short time.

We chose to obtain samples from both bone marrow and adipose tissue for the following reasons. Bone marrow is a rich source of multipotent cells, especially those associated with chondrogenic and osteogenic potential, among others [10]. Furthermore, it contains a population of very small embryonic-like stem cells, which in turn give rise to hematopoietic, mesenchymal, and endothelial progenitor cells. Adipose tissue has angiogenic properties owing to the presence of endothelial cells, pericytes, and other accessory cells, in addition to MSCs [11]. This property is beneficial in boosting the blood supply and enhancing the regeneration of tissues. Moreover, the ease of harvesting adipose tissue along with its lower immunogenic potential makes it an attractive source of a wide variety of cells. On the other hand, PRP derived from peripheral blood is a concentrate that ensures sustained release of growth factors (TGF-*β*, FGF, EGF, IGF-1, etc.), which act as nutrients and a scaffold to support proliferating and differentiating cells. Moreover, growth factors in PRP also contribute to enhanced healing through their angiogenic (VEGF) and matrix remodeling properties. A cocktail of multipotent cells and growth factors derived from these three sources provides a high concentration of the desired molecules, as well as scaffolding, thereby enhancing healing and regeneration.

The route of administration plays an important role in the degree of improvement achieved. The therapeutic effects of the MSCs and growth factors depend on their ability to migrate, adhere, and engraft into the target tissues [12]. It is known that intravenous administration of MSCs leads to their homing in lung tissue [13]. However, the presence of chemokines such as CXCR4 aid in migration of transplanted MSCs to the site of injury [14]. This is especially true in freshly isolated MSCs that express high CXCR4 when compared to cultured cells, which could possibly be attributed to the culture conditions, modified expression of surface markers, and aging/differentiation of the MSCs in culture [14]. Nonetheless, the efficacy of the MSCs does not diminish when administered intravenously [12]. On the other hand, site-specific injection (intraarticular in this case) results in high concentration of the cells in the local area, which facilitates rapid healing. Thus, we considered a combination approach by administering the cells through both routes to achieve more definitive healing.

Regeneration of tissues following sports injuries has been reported in several previous studies. Pak et al. [15] and Whitehouse et al. [16] investigated the effects of MSCs with relevance to meniscal repair and reported positive effects of the therapy. Similarly, Centeno et al. reported regeneration of meniscal cartilage in a patient who had evidence of osteoarthritis following administration of autologous MSCs [17]. A randomized controlled trial by Vangsness et al. also reported positive effects of ex vivo cultured adult human MSCs derived from the bone marrow in meniscal tissue injury [18]. The study provided evidence of meniscus regeneration and improvement in knee pain following treatment with allogeneic human MSCs. Whitehouse et al. arthroscopically implanted MSCs seeded in a collagen scaffold and reported avascular meniscal repair and long-term improvement in symptoms [16]. In the context of ACL treatment, a study reported that bone marrow-derived mesenchymal stem/stromal cells play a role in hamstring tenocyte modulation and extracellular matrix remodeling, which aids in regeneration of healthy tissues [19]. Although there are no clinical studies of direct injections of MSCs in cases of ACL injury, studies have investigated the role of MSCs and PRP in conjunction with the microfracture procedure. For example, Steadman et al. reported the repair of complete proximal ACL tear in skeletally immature athletes [20]. Microfracture reportedly leads to the formation of a blood clot and subsequent hematoma formation with bone MSCs. The possible explanation for this is migration of MSCs to the site of injury as an inherent healing mechanism, considering that MSCs are normally absent in intact ACL [21]. Likewise, Gobbi et al. evaluated the outcome after suture repair of proximal partial ACL tear combined with microfracture and injection of PRP glue at the repair site [22]. They reported that the bone MSCs and PRP might act as the source of precursor cells and growth factors, thereby resulting in enhanced clinical outcomes. The middle-term results of the study revealed that 78% of the 50 recruited athletes could return to their sports activities.

The results of our study corroborate with those previously reported in the literature regarding regeneration of meniscal and cruciate ligament tissue. We considered a noninvasive, only injection approach, as the patient was unwilling to undergo surgery. Considering the favorable results achieved, it may be considered that the outcomes depend on targeted delivery of the required cells and growth factors. Nonetheless, maintenance of the outcomes in the present case needs to be evaluated through long-term follow-up. Moreover, the therapeutic approach should be decided on a case-to-case basis, depending on the extent and severity of injury, to achieve optimal results.

Thus, cell- and growth factor-based therapy can be considered a safe and effective therapeutic modality in the treatment of meniscal tears and cruciate ligament injury. Being minimally invasive, and considering the autologous source of cells, the therapy can be performed in young and old individuals alike, irrespective of the presence/absence of comorbid conditions. The autologous source also limits the ethical, legal, and safety/logistic issues associated with obtaining allogeneic stem cells. However, an appropriate treatment plan describing the advantages and possible risks of autologous cell-based therapy must be formulated and be approved by the relevant ethics committees, prior to initiation of treatment.

A major limitation of our study is the small sample size. This was the first case of meniscal and ACL injury treated using a regenerative approach at our center. A robust case-control, randomized study on a larger sample size is being planned to validate the outcomes and reliability of autologous cell-based therapy in meniscal injury, with or without cruciate ligament injury. Future studies should focus on long-term follow-up in large populations across age groups, through specific routes of administration for more precise knowledge about the effect of cell-based therapy in meniscal tissue and cruciate ligament regeneration.

## Figures and Tables

**Table 1:** Grading of meniscal injury.

Grade	Imaging findings
0	Normal intact meniscus
I	Intrasubstance globular-appearing signal not extending to the articular surface
II	Linear increased signal patterns not extending to the articular surface
III	Abnormal signal intersects the superior and/or inferior articular surface of the meniscus, an arthroscopically confirmable tear

**Table 2:** Grading of anterior cruciate ligament injury.

Grade	Imaging findings
I	Mild damage; ligament is slightly stretched but is able to keep the joint stable
II	Partial tear; stretched ligament to the point that it becomes loose
III	Complete tear; ligament is split into two and the knee joint is unstable

**Table 3:** Pre- and post-treatment MRI findings of the patient.

Pre-treatment	Post-treatment
High-grade tear of ACL	Intersubstance tear of ACL only near femoral attachment
Grade III complex tear of posterior horn of medial and lateral meniscus	Grade II tear in the posterior horn of the medial and lateral meniscus
Mild knee joint effusion	Mild collection seen around knee joint
Marrow edema and subcortical depression in the medial tibial plateau	No edema in medial tibial plateau
